# HIV incidence and behavioral correlates of HIV acquisition in a cohort of injection drug users in St Petersburg, Russia

**DOI:** 10.1097/MD.0000000000005238

**Published:** 2016-11-04

**Authors:** Andrei P. Kozlov, Roman V. Skochilov, Olga V. Toussova, Sergey V. Verevochkin, Tatiana V. Krasnoselskikh, Sergey V. Malov, Alla V. Shaboltas

**Affiliations:** a Biomedical Center; b Peter the Great St Petersburg Polytechnic University; c St Petersburg State University; d Pavlov First Saint Petersburg State Medical University; e St Petersburg Electrotechnical University, St Petersburg, Russia.

**Keywords:** cohort study, HIV incidence, IDU

## Abstract

The aim of the project was to study human immunodeficiency virus (HIV) incidence, sociodemographic and behavioral correlates of HIV acquisition among injection drug users (IDUs).

A total of 717 IDUs were recruited, tested, and counseled for HIV-1; 466 HIV-negative participants were enrolled and followed-up at 6 and 12 months. Sociodemographic and behavioral data were collected during each study visit. The association of sociodemographic and behavioral factors to HIV-1 incidence was assessed.

During the 9-month recruitment period, 717 IDUs were screened and 466 participants were enrolled. HIV-1 prevalence at baseline was 35%. Most enrolled subjects were young (median age 30), male (75%), injected heroin in the previous 3 months (86%), about 50% had shared syringes and other paraphernalia, and 44% had unprotected sex in the last month. The retention rate at the 12-month follow-up was 72% and the adjusted retention rate was 88%. The HIV incidence rate was 7.2/100 person-years. HIV incidence was significantly associated with specific drug risk behaviors, including injecting the mixture of heroin and psychostimulants, the frequency of injecting in groups with other people, and having more drug dealers.

The St Petersburg IDUs cohort demonstrates one of the highest HIV incidence rates in the world. In 2004 to 2006, the HIV incidence was 4.5, in 2005 to 2007—19.6, and in 2008 to 2009—7.2/100 person-years. The peak of HIV epidemic among IDUs in St Petersburg, as determined by 3 independent cohort studies, was in 2006 to 2007. Interventions targeting IDUs with long experience of heroin injection and high levels of injection risk behaviors are urgently needed.

## 1 Introduction

Injection drug use became a major factor of the human immunodeficiency virus (HIV)-1 epidemic in Russia since 1995 to 1996.^[1]^ Among 907,607 HIV cases officially registered by the Federal AIDS Center in Russia by the end of 2015, more than 50% are still attributed to injection drug use.^[2]^ The dramatic increase of new HIV cases associated with unsafe injection behavior since late 1990s is due to numerous factors—political, economic, and social changes, which were common for all countries in Central and Eastern Europe during the transition period.^[3]^ The significant increase in drug trafficking routes from Afghanistan and other South Asian countries through Russia to Europe, and the introduction of “pure” heroin which replaced the homemade opiates has become the major driving factor for the HIV epidemic, spread by injecting drug use.^[4–6]^

In St Petersburg, the second largest city in Russia, the total number of registered HIV cases at the end of 2015 reached 57,171 according to official data from the Russian Federal AIDS Center.^[2]^ Despite the increasing proportion of HIV cases attributed to sexual transmission, unsafe injection practices still play the most significant role in HIV transmission in St Petersburg. In 2010, about 76% of new HIV cases were due to unsafe injection behaviors.^[7]^

In the first longitudinal HIV incidence study conducted among injection drug users (IDUs) in St Petersburg as a part of HIV Prevention Trial Network, the HIV prevalence rate was 30% and the HIV incidence rate was 4.5/100 person-years.^[5,6]^

Other studies conducted among IDUs in St Petersburg in later years demonstrated the increase of HIV epidemic in this population. In a randomized control trial of Russian IDU peer network HIV prevention intervention, the HIV prevalence rate in 2005 was 44% at baseline^[8,9]^ and the incidence rate was 19.6/100 person-years.^[10]^ In a study on Sexual Acquisition and Transmission of HIV—Cooperative Agreement Program, HIV prevalence rate reached 50% in 2007.^[11]^ The incidence rate estimated by retrospective cohort analysis was 14.1/100 person-years and results of BED EIA (Calypte HIV-1 BED Incidence EIA test) estimates were even higher and reached 25.5/100 person-years in this sample.^[12]^

The main objectives of this study were to determine the HIV prevalence and incidence in 2008 to 2010 and to identify behavioral and sociodemographic determinants of HIV acquisition among IDUs in St Petersburg.

## 2 Methods

### 2.1 Participant recruitment

The main eligibility criteria for the participants to be enrolled in this study included the following: HIV-negative status, experience of injecting drugs or sharing injecting paraphernalia with another person at least once within the previous 6 months, and age 18 or older (the age limit of 18 is a standard for participation in biomedical studies in Russia without parental consent).

Prior to the initiation of the study, the protocol and informed consent forms were approved by the Institutional Review Board at the Biomedical Center in St Petersburg. The recruitment, enrollment, and follow-up study visits were conducted at the clinic of the Biomedical Center. Social network approach (“snowball”) was used as the main recruitment strategy for this study. This approach has demonstrated the efficiency in the previous IDU studies conducted in St Petersburg.^[6,10]^

### 2.2 Data collection

All study procedures, including eligibility confirmation and obtaining 2 informed consents (for the participation in the study and blood samples storage), were conducted at the research site in accordance with the detailed study protocol. The screening visit for participants included pretest counseling and HIV-1 testing using enzyme immunoassay with confirmatory Western blot analysis. The administration of a risk-assessment interview preceded every blood test and included questions on sociodemographic and behavioral characteristics, such as drug injecting practices (types of drugs, frequency of using and sharing experience), sexual behaviors, alcohol use, and other health-related issues. Participants were instructed to return in 14 days to obtain test results and receive post-test counseling. HIV-negative participants were enrolled into the study and received their follow-up visit schedule. HIV-positive participants were counseled and referred to medical and psychological primary care available at St Petersburg City AIDS Center.

The follow-up visits were scheduled at 6 and 12 months with a 45-day window period (15 days prior and 30 days after the scheduled date for the study visit) and included the same study procedures as the baseline visit. After each study visit, participants received gifts (food vouchers or mobile phone cards) and subway tokens as incentives. The value of incentives was around 600 roubles (approx. $20 in 2008–2009), determined by St Petersburg Institutional Review Board to be noncoercive for participants.

### 2.3 Statistical analysis plan

All case report forms were entered into the database using PHP/MySQL/Apache technologies that were especially designed for this particular study at the Biomedical Center. All analyses were performed using SAS software (version 9.1) and R software (version 2.12.0). The target sample size was 450 to provide a half-width of 3% for the 95% confidence interval (CI) for HIV incidence based on a 12-month retention rate of 80% and a true HIV incidence rate of 8%. Single- and multi-factor analyses of the sociodemographic and behavioral factors to incidence of HIV-1 infection were conducted using an exponential parametric model for interval censored data.^[13,14]^ Hazard rates and significant conclusions were verified using Cox proportional hazards models. Associations at *P* ≤ 0.10 were entered into the additive multiple Cox regression models and we selected the best one by using akaike information criterion to report.

## 3 Results

### 3.1 Screening and enrollment

During the screening period between July 2008 and May 2009, 717 IDUs met the eligibility criteria and expressed interest to participate in the study. Among them, 490 (68%) acquired the information from the study and have been referred by another study participant. Of the 714 participants who went through all screening procedures and signed informed consents for the study at baseline, 248 (35%) were tested HIV-positive; 466 (65%) HIV-negative participants were enrolled in 1-year follow-up study.

### 3.2 Baseline characteristics

Table 1 provides data on the sociodemographic characteristics of the enrolled individuals. The cohort of HIV-negative participants was mostly male (350; 75%), had a median age of 30.8, single (303; 65%), had completed at least a secondary education (420; 90%), was unemployed or partly employed (350; 75%), lived with relatives, parents, or friends (406; 87%), and had an average monthly income of <24,000 roubles (approx. $800 in 2008–2009) (370; 79%).

**Table 1:**
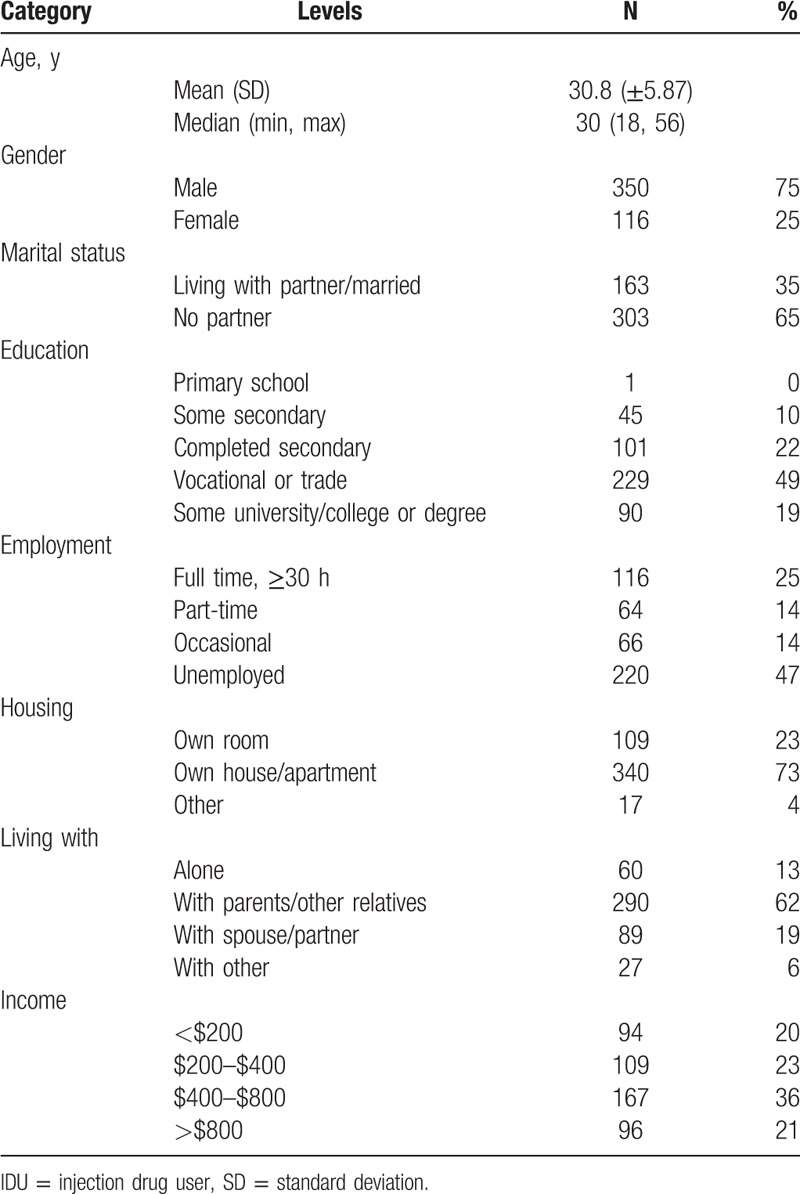
Sociodemographic characteristics of human immunodeficiency virus negative IDU cohort (N = 466).

Table 2 describes the drug risk behaviors in the 3 months preceding the baseline. For HIV-negative cohort heroin was the most common drug of injection (86%), 19% injected methadone, 13% injected amphetamines, and 4% injected ephedrine-based psychostimulants. The percentages sum to more than 100% because individuals may choose more than 1 type of drug. The median frequency of drug injecting was 3 to 6 times/wk; 67% demonstrated different types of sharing risk behaviors including sharing needles (30%); 4% injected drugs sharing equipment with a HIV-positive person.

**Table 2:**
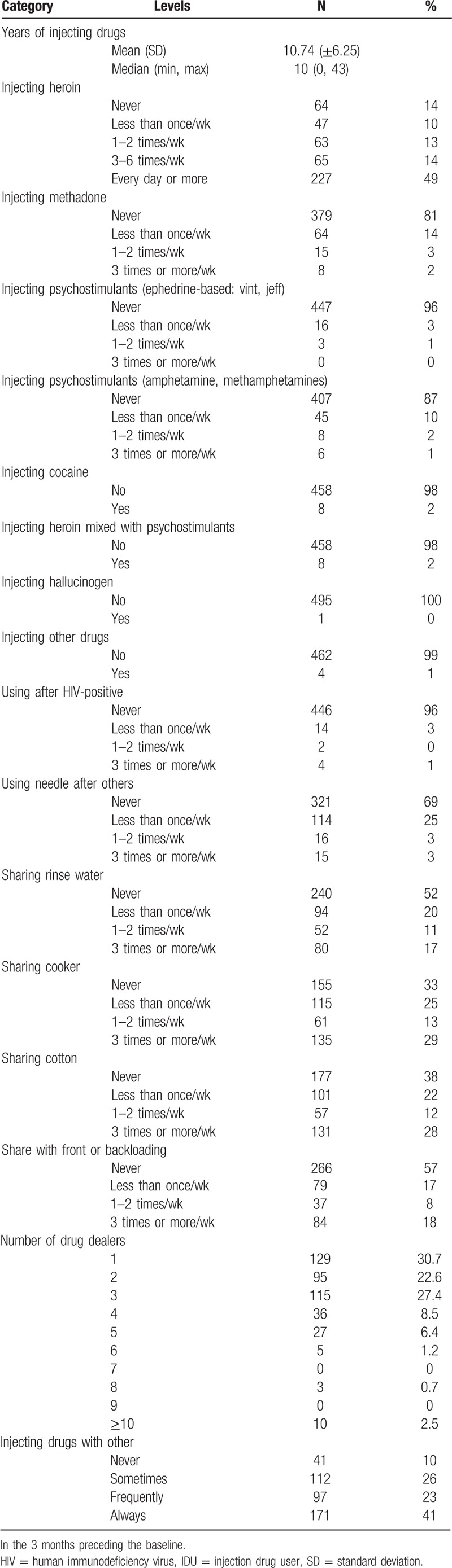
Baseline drug risk behaviors of HIV-negative IDU cohort (N = 466).

Of HIV-negative participants, 76% were sexually active in the 3 months preceding the baseline, 82% of them had a primary sexual partner with 48% of these partners being IDUs; 44% reported having unprotected sex in the last month prior to baseline. Data on sexual risk behaviors and health status are presented in Table 3.

**Table 3:**
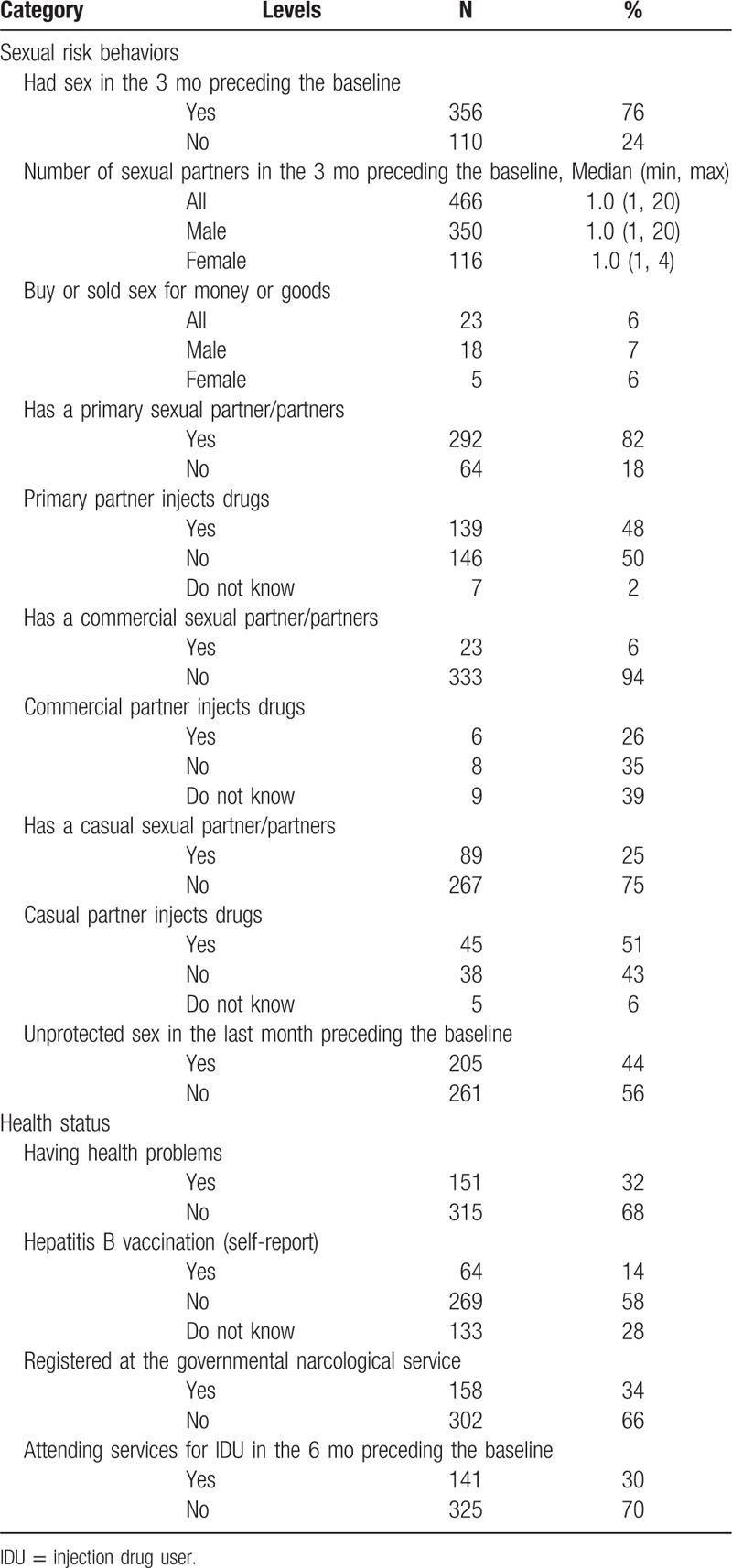
Sexual risk behaviors and health status of human immunodeficiency virus negative IDU cohort (N = 466).

### 3.3 Retention

Among the 466 subjects, who were enrolled in the follow-up study, 334 (72%) had 12-month visit or HIV seroconverted during the follow-up period. Among the 132 subjects lost to follow-up, 20 (15%) had died, 54 (41%) were incarcerated, 4 (3%) were hospitalized in rehabilitation programs, 6 (4.5%) stopped using drugs and quit participation, 6 (4.5%) quit participation, but did not stop using drugs, 17 (13%) moved outside of the city, and 25 (19%) stopped participating for other reasons. Of 466 subjects who attended at least 2 visits, 380 (72%) were enrolled into the incidence analysis.

### 3.4 Incidence of HIV-1

During 12-month follow-up period, 28 participants were seroconverted. The HIV incidence rate was 7.2/100 person-years. Results of single-factor analysis identifying the sociodemographic and behavioral correlations with HIV incidence are shown in the Table 4 .

**Table 4:**
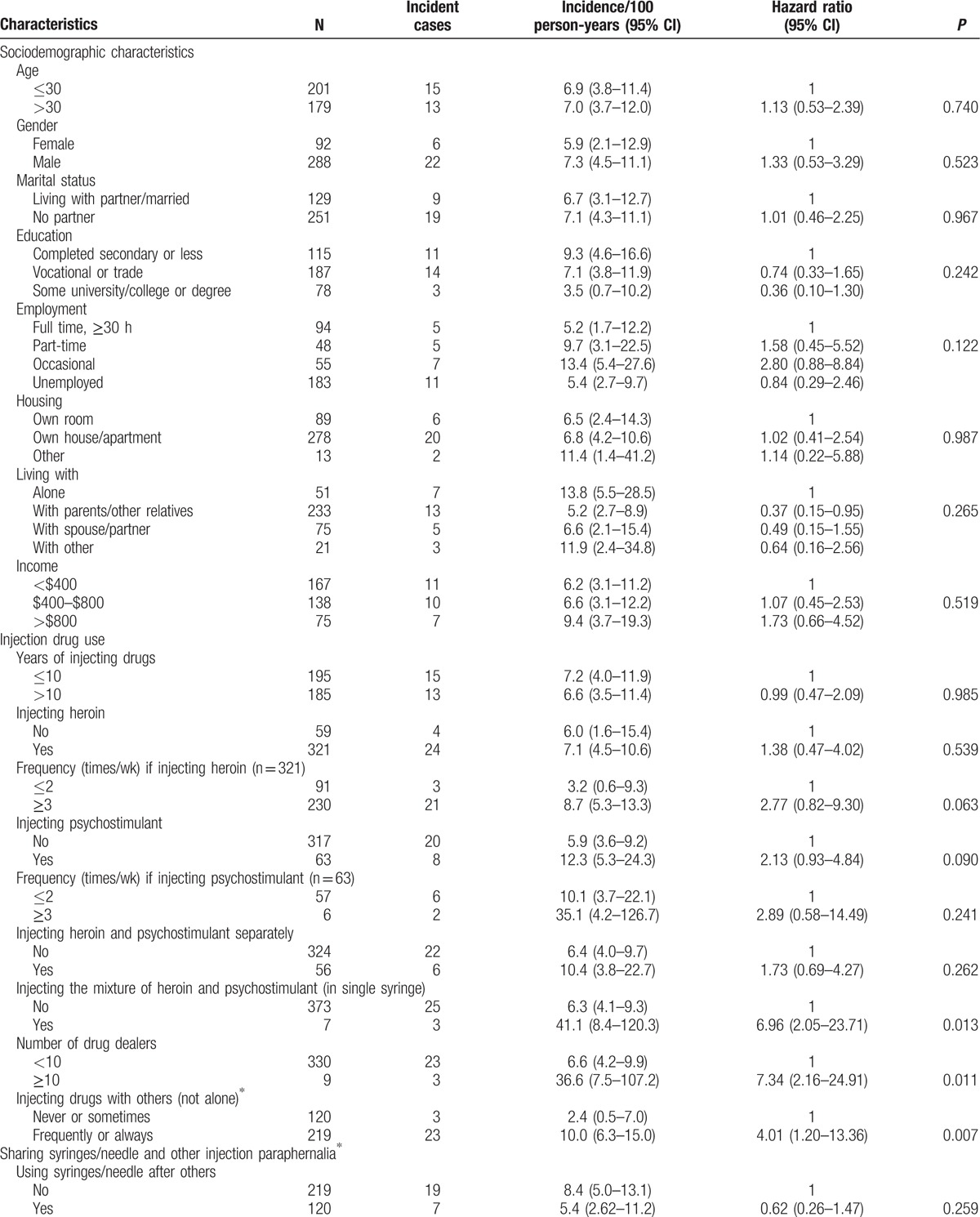
Sociodemographic and behavioral characteristics associated with incident human immunodeficiency virus infection in univariate analysis (N = 380).

**Table 4 (Continued):**
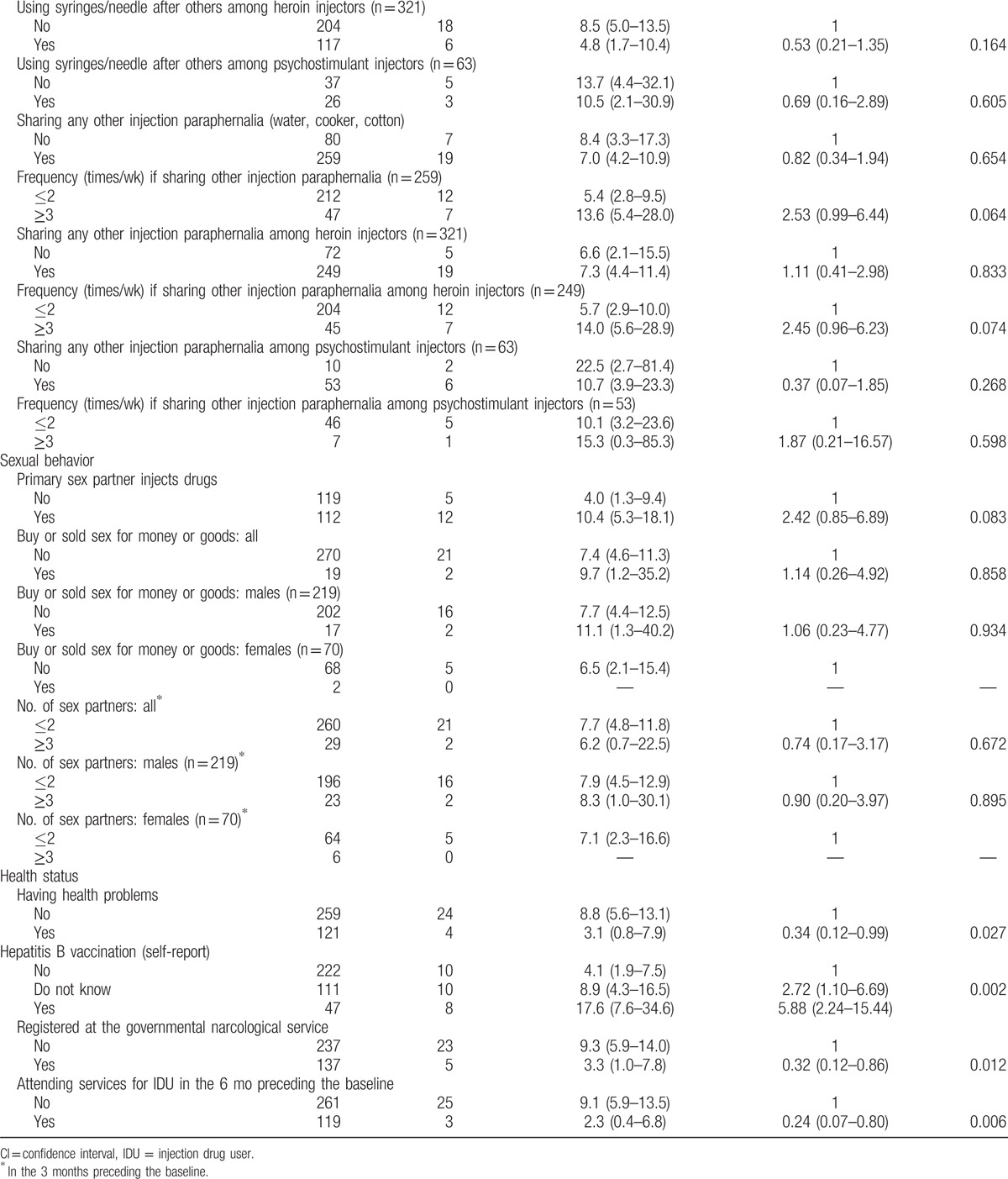
Sociodemographic and behavioral characteristics associated with incident human immunodeficiency virus infection in univariate analysis (N = 380).

There were no significant associations found between the HIV-1 incidence rate and sociodemographic characteristics, such as gender, age, marital status, employment, housing, and monthly income. The single-factor analysis revealed several drug risk behavior factors associated with HIV incidence. The HIV incidence rate among IDUs who injected the mixture of heroin and psychostimulants was significantly higher in comparison with those who did not practice injecting such a mixture (hazard ratio [HR], 6.96; 95% CI, 2.05–23.71; *P* = 0.013). Those IDUs who injected drugs more frequently with others (not alone) in the 3 months preceding the baseline had significantly higher risk of HIV acquisition (HR, 4.01; 95% CI, 1.20–13.36; *P* = 0.007). Buying drugs from 10 and more drug dealers during the 3 months preceding the baseline was also identified as significant factor associated with HIV acquisition (HR, 7.34; 95% CI, 2.16–24.91; *P* = 0.011). Using “number of drug dealers” as a numeric variable in Cox linear regression model also showed a good fit and displayed the similar significance (HR, 1.23; 95% CI, 1.07–1.41; *P* = 0.014).

Additionally, HIV incidence was associated with current health status. Three criteria related to health status were significantly associated with lower HIV risk—attending services for IDUs in the 6 months preceding the baseline (HR, 0.24; 95% CI, 0.07–0.80; *P* = 0.006), having health problems (HR, 0.34; 95% CI, 0.12–0.99; *P* = 0.027), and being registered at the governmental narcological service (HR, 0.32; 95% CI, 0.12–0.86; *P* = 0.012). IDUs who reported hepatitis B vaccination demonstrated significantly higher levels of HIV risk behaviors (HR, 5.88; 95% CI, 2.24–15.44; *P* = 0.002).

In the multi-factor analysis, subjects who injected the mixture of heroin and psychostimulants, had 10 and more drug dealers and reported hepatitis B vaccination continued to be associated with HIV incidence; frequently or always injecting drugs with others (not alone) in the 3 months preceding the baseline and attending services for IDU in the 6 months preceding the baseline displayed significance are very close to 5% level (Table 5). Due to strong association between the variables “Attending services for IDU in the 6 months preceding the baseline” and “Having health problems” (*P* = 5.2 × 10^−4^), we fitted another model using these factors simultaneously (including interactions). The combined factor displayed significant association in the multivariate model (*P* = 0.024). The results for the others 4 factors involved into the multivariate analysis were very similar.

**Table 5:**
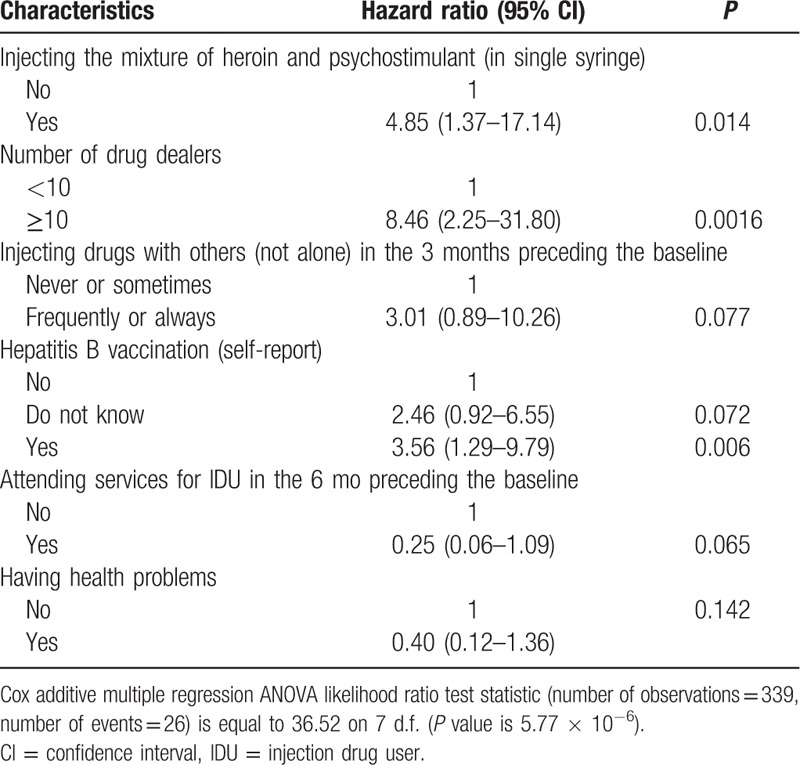
Behavioral factors associated with incident human immunodeficiency virus infection by Cox additive multiple regression model (N = 339).

## 4 Discussion

Extremely high levels of HIV incidence in the current IDU cohort (7.2/100 person-years) express the lack of effective HIV-prevention efforts focused on this population and implemented at the governmental level in Russia. This conclusion is supported by our data that shows significant differences in HIV incidence rates between participants who applied for governmental services for IDUs and those who did not.

In 2008 to 2009, heroin was the main drug of abuse in the current IDU cohort, similar to the IDU cohort recruited in 2002. Nevertheless, the types of primarily used psychostimulants changed since 2004. In the 2002 to 2004 IDU cohort, the primarily used psychostimulants were mostly ephedrine-based, which determined the greater HIV risks, due to the frequency of injecting and having multiple sexual partnerships in a short-time period.^[5,6]^ In 2008 to 2010, ephedrine-based psychostimulants were replaced by amphetamines and methamphetamines.

Single-factor analysis revealed several behavior factors associated with HIV incidence. Mixture of heroin and psychostimulants could reflect either a higher demand of sensation-seeking or the usage of whatever was currently available. Buying drugs from 10 and more drug dealers during the 3 months preceding the baseline could lead to higher HIV risks, due to unstable and unknown environments and substance quality. Participants who reported hepatitis B vaccination demonstrated the higher level of HIV incidence. Perhaps, those participants considered themselves at lower HIV risk due to vaccination and practiced higher levels of unsafe behaviors. All 3 mentioned behavioral factors were still significant in multifactor analysis. Three criteria related to health status were significantly associated with lower HIV risk—attending services for IDUs in the last 6 months, having health problems, and being registered at the governmental narcological service. These data demonstrate the importance of state healthcare establishments to conduct HIV testing accompanied with counseling for IDUs, to provide them with test results, and to create the environment in facilities for follow-up activities that would lack stigma, promote friendly communication, and facilitate desire for IDUs to return to this facility or to a certain specialist.

There are several limitations in this study. First, the sample consists of opioid users mostly and therefore received findings may not reflect situation with HIV acquisition among injectors of other substances. Second, the study was conducted in St Petersburg and its findings may not be applicable to other regions of Russia. Third, 28% of the sample was lost for follow-up, thus decreasing opportunities to reveal other important factors associated with seroconversion.

However, the high HIV incidence among IDU and received results on factors associated with seroconversion, confirm the importance of HIV prevention efforts among IDU population and suggest the necessity of health care structures being involved into HIV prevention programs targeting IDU.

## Acknowledgments

The authors acknowledge the dedication the study team members, including social workers, counselors, physicians, nurses, lab staff, office managers, and drivers whose hard work and enthusiasm played the crucial role in the successful implementation of this study.
